# On the investigation of non-specific effects of BCG: Interpreting global vaccine data

**DOI:** 10.1016/j.ebiom.2021.103321

**Published:** 2021-04-12

**Authors:** Ane Bærent Fisker, Kristoffer Jarlov Jensen

**Affiliations:** a Bandim Health Project, OPEN, University of Southern Denmark, Copenhagen, Denmark; b Bandim Health Project, Bissau, Guinea-Bissau; c Copenhagen Phase IV Unit, Center for Clinical Research and Prevention, Frederiksberg and Bispebjerg Hospital, Frederiksberg, Denmark

That Bacillus Calmette Guerin (BCG) vaccination may alter the outcome of subsequent non-tuberculosis infectious challenges, has already been demonstrated in many studies [1] including randomised trials with clinically important outcomes both among neonates [2] and more recently also in adults [3]. Another, but related manifestation of non-specific effects of BCG is the potentiation of immune responses to unrelated vaccines. In the present article of *EBioMedicine* [4], Esther Broset and colleagues investigate if BCG affects immunity induced by diphtheria-tetanus-pertussis vaccines (DTP). Broset and colleagues present interesting experimental data showing that in mice, the humoral and Th1 responses to diphtheria-tetanus-acellular pertussis vaccine (DTaP) are augmented by giving BCG (or a new tuberculosis vaccine, MTBVAC) vaccine prior to DTaP vaccine(s).

Based on the finding that prior BCG/MTBVAC may alter the immunity towards DTP, the authors also investigate the potential implications for humans. They do so by correlating vaccination programmes and country level cases of pertussis. Interestingly, they find that during the past 5 years, countries with BCG vaccine in their vaccination programme have a lower reported burden of pertussis infections than countries without BCG in the vaccination schedule. They find this pattern, both in global data and in strata of the global data defined by geography and income level. This analysis, however, has some pitfalls, as also acknowledged by the authors. For that part, we would like to commend the authors for attempting to address confounding through triangulation, stratified analyses and investigation of alternative control outcomes. A potential confounder is that the disease surveillance quality may also be correlated to the BCG vaccination programme: countries having phased out BCG due to low tuberculosis incidence may also have better disease surveillance. To that end the authors exclude countries that have no reports of pertussis for the past 5 years from the analysis.

As an additional approach, the authors restrict the analysis to European data only, upon which they reassuringly reach the same conclusion. The efforts to control shortcomings of the outcome (pertussis incidence) data implicitly highlight that there is a vast scope for improvement in the outcome data. Ongoing efforts to strengthen the global surveillance capacity [5] will hopefully contribute to better data on pertussis and other vaccine preventable diseases in the future.

To further triangulate the question, the authors investigate whether the annual reported rate of pertussis cases increases when BCG vaccination is interrupted (due to shortages) or stopped. While they present some support that there may be increases in pertussis cases when BCG coverage was low or phased out, they did not find the same pattern when seeking to correlate country level BCG vaccination coverage or recommended age at BCG vaccination (at birth vs later) with pertussis incidence. They speculate that this may be because the coverage rates are generally very high, and therefore that there is little variation due to lack of heterogeneity in the exposure.

It is our experience, however, that the reported country level data on vaccine coverage and scheduled vaccination age is not necessarily a good indicator of the actual age of vaccination [6]. In the present work, what looks as homogeneous coverage rates may masks substantial disparities in timeliness of BCG vaccination. A reported >90% coverage and BCG being recommended at birth, does not mean that 90% of infants are vaccinated at birth. There are substantial delays in BCG vaccination in many settings. The majority of the sub-Saharan African countries recommend BCG at birth, but most children receive the vaccine after the neonatal period [7]. Similar delays occur in Bangladesh [8]. However, with coverage assessed only at 12 months of age, this delay remains hidden [9]. Hence, a programme with BCG scheduled at birth, does not necessarily mean that the vaccine is given at birth.

While we appreciate the present investigation as an important and relevant study on the potential non-specific effects of BCG, evaluated on its own, the ecological data does not provide strong evidence that BCG affects the susceptibility to pertussis.

With growing acceptance that BCG vaccines in addition to protecting against tuberculosis may prevent other infections, better statistics on BCG coverage may serve several purposes. They could shed light on how the BCG vaccination policy is actually being implemented and thereby how the programme could be strengthened, but also allow investigation of whether heterogeneity in the implementation is associated with/potentially affects measures of health outcomes.

In conclusion, the triangulation exercise undertaken by the authors here does bring us further, and investigates the potential impact of BCG vaccination for a particular pathogen. Whether a potential effect is mediated by BCG in itself or an interaction between BCG and DTP vaccine cannot be concluded based on the ecological data. But it does suggest that removing BCG when tuberculosis is under control, may not be a wise decision. Replacing BCG by other vaccines, such as MTBVAC would have to be investigated in trials evaluating both tuberculosis and other health outcomes.

## Contributors

ABF wrote the first draft of the manuscript. Both authors edited and approved the final manuscript.

## Declaration of Competing Interest

The authors declare no conflict of interest.
